# Impact of Environmental Conditions on the Concentrations of Trichothecenes, Their Glucosides, and Emerging *Fusarium* Toxins in Naturally Contaminated, Irradiated, and *Fusarium langsethiae* Inoculated Oats

**DOI:** 10.3390/toxins16040166

**Published:** 2024-03-22

**Authors:** Abimbola Oluwakayode, Brett Greer, Julie Meneely, Franz Berthiller, Rudolf Krska, Angel Medina

**Affiliations:** 1 Applied Mycology Group, Environment and AgriFood Theme, Cranfield University, College Rd., Wharley End, Bedford MK43 0AL, UK; abimbola.oluwakayode@cranfield.ac.uk; 2 Institute for Global Food Security, National Measurement Laboratory: Centre of Excellence in Agriculture and Food Integrity, Queen’s University Belfast, 19 Chlorine Gardens, Belfast BT9 5DL, UK; brett.greer@qub.ac.uk (B.G.); j.p.meneely@qub.ac.uk (J.M.); rudolf.krska@boku.ac.at (R.K.); 3 The International Joint Research Centre on Food Security (IJC-FOODSEC), 113 Thailand Science Park, Pahonyothin Road, Khong Luang 12120, Thailand; 4 Institute of Bioanalytics and Agro-Metabolomics, Department of Agrobiotechnology IFA-Tulln, University of Natural Resources and Life Sciences, Vienna, 3430 Tulln, Austria; franz.berthiller@boku.ac.at

**Keywords:** T-2 toxin, HT-2 toxin, diacetoxyscirpenol, deoxynivalenol, masked mycotoxins, LC-MS/MS, oat grains

## Abstract

Trichothecenes produced by *Fusarium* species are commonly detected in oats. However, the ratios of the concentrations of free trichothecenes and their conjugates and how they are impacted by different interacting environmental conditions are not well documented. This study aims to examine the effect of water activity (0.95 and 0.98 a_w_) and temperature (20 and 25 °C) stress on the production of T-2 and HT-2 toxins, deoxynivalenol and their conjugates, as well as diacetoxyscirpenol (DAS). Multiple mycotoxins were detected using liquid chromatography–tandem mass spectrometry from 64 contaminated oat samples. The highest concentrations of HT-2-glucoside (HT-2-Glc) were observed at 0.98 a_w_ and 20 °C, and were higher than other type A trichothecenes in the natural oats’ treatments. However, no statistical differences were found between the mean concentrations of HT-2-Glc and HT-2 toxins in all storage conditions analysed. DAS concentrations were generally low and highest at 0.95 a_w_ and 20 °C, while deoxynivalenol-3-glucoside levels were highest at 0.98 a_w_ and 20 °C in the naturally contaminated oats. Emerging mycotoxins such as beauvericin, moniliformin, and enniatins mostly increased with a rise in water activity and temperature in the naturally contaminated oats treatment. This study reinforces the importance of storage a_w_ and temperature conditions in the high risk of free and modified toxin contamination of small cereal grains.

## 1. Introduction

*Fusarium langsethiae,* originally referred to as a ‘powdery *Fusarium poae*’, is a major producer of the type A trichothecenes T-2 toxin (T-2) and HT-2 toxin (HT-2), initially discovered in Norwegian cereals [1,2]. It is characterized by spores morphologically similar to *Fusarium poae,* with a similar mycotoxin profile to *Fusarium sporotrichioides* [3]. The occurrence of *Fusarium langsethiae* and its mycotoxins in a wide range of cereals and cereal products such as oats, wheat, and barley has been extensively examined [4,5,6,7,8,9].

Diacetoxyscirpenol (DAS), mainly produced by *Fusarium langsethiae* and mostly found in cereal grains, has been assessed for its toxicity to humans and animals by the European Food Safety Authority (EFSA) [10]. DAS, also called anguidine, belongs to group A of trichothecenes, having a similar structure and toxicity to T-2 toxin, causing lymphoid necrosis, haematotoxicity, and gastrointestinal toxicity [11,12]. Considered a severe threat to human, animal, and plant health, DAS is enlisted for research in the ‘Select Agents and Toxins’ list of the Centers for Disease Control and Prevention, United States [13]. DAS has been found to reduce feed intake and body weight with other cytotoxic effects such as oral lesions in broiler breeders fed with a DAS-contaminated diet [14]. Its occurrence in cereal and cereal-based foods is not well documented.

Deoxynivalenol (DON) has been found in oats and was reported to be mainly produced by *F. graminearum,* which is the main causative agent of *Fusarium* head blight (FHB) in small cereal grains, especially in temperate regions [6,15,16]. Unlike *F. langsethiae*, it is a strong pathogen that can penetrate plant cell walls, causing aggressive colonisation [17,18]. DON has been reported to co-occur with its glucoside, DON-3-G, in cereal-based products and with acetylated forms in oats [19,20,21]. However, these studies did not investigate the impact of storage conditions on the concentrations of these mycotoxins in oats.

As a major contaminant of oats, T-2 toxin can be rapidly metabolized to HT-2 toxin [22]. The effects of both toxins include a weakened immune system, growth retardation in exposed animals, and cytotoxicity in the human small intestine, which are well detailed in the JECFA report on HT-2 and T-2 toxins and other related studies [23,24,25]. Due to their toxicity, indicative levels for the sum of T-2 and HT-2 (μg/kg) have been set by the European Commission at 1000 μg/kg in unprocessed oats (with husks), with a tolerable daily (TDI) intake of 0.02 μg/kg of body weight per day [26].

T-2 and HT-2 can be metabolized to their modified forms, such as HT-2-glucoside, T-2-glucoside, 3′-hydroxy-T-2, T-2 tetraol, and 3′-hydroxy-T-2 triol [23,27]. While these metabolites are less toxic, the glucosides can be converted back to their native form in the mammalian gastrointestinal tract to regain their toxicity [27]. These modified forms are not readily detected by conventional analytical techniques, making it difficult to predict the total mycotoxin levels in harvested grains [28]. Grains that seem to be clean or healthy may be contaminated with high levels of mycotoxins and their modified forms due to the frequent occurrence of *Fusarium* species in small-sized cereal grains without visible traces of infection [29,30]. It is therefore important to understand *Fusarium* infection either pre- and/or post-harvest and examine how environmental factors influence mycotoxin contamination of grains by *Fusarium langsethiae*.

A storage temperature of 25 °C and 0.995 a_w_ was reported as the optimum temperature for the growth of two strains of *Fusarium langsethiae* on oat-based media [31]. The highest concentrations of T-2 and HT-2 in oat grains in situ were reported at 25 °C in the wettest condition of 0.97 a_w_ by Mylona and Magan, [32]. However, while these studies showed the growth of *Fusarium langsethiae* and the production of T-2 and HT-2 in oats in vitro and in situ, they have not shown how their concentrations vary alongside their conjugates in stored oats under different environmental conditions. Therefore, as maximum levels for T-2 and HT-2 toxins would be set in the year 2024 by the European Union Commission, it is imperative to examine if different environmental conditions influence free T2, HT-2, and their conjugated forms in stored oat grains.

The objectives of this study were to examine the impact of storage conditions, namely water activities of 0.95 a_w_ and 0.98 a_w_ and temperatures of 20 °C and 25 °C, on (a) the concentrations of free T-2 and HT-2, DON, and their respective glucosides and (b) the concentrations of DAS and other emerging mycotoxins in both naturally contaminated and irradiated oat grains inoculated with *Fusarium langsethiae* to ascertain any potential increases in toxicity in the oat grains.

## 2. Results

### 2.1. Method Validation Performance in the Oat Matrix

The multi-analyte extraction methodology was based on a dilute-and-shoot approach. The extraction efficiency (RE) of each analyte was within the acceptable range (70–120%), according to the amended guideline set by European Commission regulation No. 2021/808/EC [33], and showed good relative standard deviation (RSD) values of <15%. The goodness of fit of the calibration curve for each analyte was acceptable, with r^2^ values (coefficient of determination) of >0.990. The relative standard deviation of the within-laboratory reproducibility (RSD_WLR_), the matrix effect or the signal suppression/enhancement (SSE), and the apparent recovery (R_A_%) were calculated from the average of 75 replicates of the five different lots of oats spiked in quintuplicate across three separate days. The trueness and precision represented by the relative standard deviation (RSD%) of the method were satisfactory (<14%) for the validated analytes, except for nivalenol (NIV). The limit of detection (LOD) and limit of quantitation (LOQ) for the analytes ranged from 1 to 19 µg/kg and 3 to 63 µg/kg, respectively, which were lower than the minimum acceptable levels for the regulated mycotoxins in unprocessed oats [34]. The mean values of the R_A_, RE, RSD, LOD, and LOQ for each analyte are shown in Table 1. The relative expanded measurement uncertainties for each analyte are shown in Table S1 in the Supplementary Materials.

### 2.2. Initial Mycotoxin Concentrations of Oats

The initial average concentrations of DON, T-2, HT-2, and HT-2 glucoside in the naturally contaminated oats before the storage experiment were 26.9 ng/g, 61.2 ng/g, 92.2 ng/g, and 32.8 ng/g, respectively. A one-way ANOVA showed significant differences among the concentrations of T-2, HT-2, and HT-2-Glc concentrations (*p* < 0.05).

### 2.3. Impact of a_w_ and Temperature on the Concentrations of T-2, HT-2, and HT-2-Glc in All Oat Treatments

The concentrations of HT-2-Glc were 1.5 times higher than the concentrations of HT-2 toxin at 0.95 a_w_ at 20 °C in the naturally contaminated oats (control samples). However, there were no significant differences (*p* > 0.05) in their concentrations. T-2 toxin, HT-2 toxin, and HT-2-Glc concentrations were below the limit of detection (LOD) or limit of quantitation (LOQ) at 25 °C for both water activity levels examined.

Despite *F. langsethiae* inoculation in the oats, toxins levels were below the LOD or LOQ at all storage conditions except at 0.98 a_w_ at 20 °C, with HT-2-Glc concentrations of 37 ng/g. A similar trend was observed in the irradiated oat control samples.

In the irradiated inoculated oats, HT-2-Glc levels significantly increased with a rise in water activity and decreased with temperature rise, with concentrations higher than other oat treatments. HT-2-Glc was highest at 0.98 a_w_ at 20 °C. A similar trend was observed for the concentrations of HT-2 toxin in most storage conditions except at 0.98 a_w_ at 25 °C, where an increase in concentrations was observed, with the highest concentrations of 7167 ng/g. T-2 concentrations were significantly higher than HT-2 and HT-2-Glc concentrations at 0.95 a_w_ at both temperatures. However, at 0.98 a_w_ and at both 20 and 25 °C, T-2 concentrations were not significantly different (*p* > 0.05) from HT-2-Glc concentrations. HT-2 levels were significantly higher than T-2 concentrations at 0.98 a_w_ and 25 °C, and no significant differences (*p* > 0.05) exist in their concentrations at 0.98 a_w_ and 20 °C, as shown in Table 2.

The *p* values of the statistical differences in the concentrations of T-2, HT-2, and HT-2-Glc under different storage conditions in all oat treatments are shown in Tables S2 and S3 in the Supplementary Materials.

### 2.4. The Effect of the Interactions of Water Activity, Temperature (T), and Treatments on the Percentage Concentration Ratios of HT-2-Glucoside

**Figure 1:**
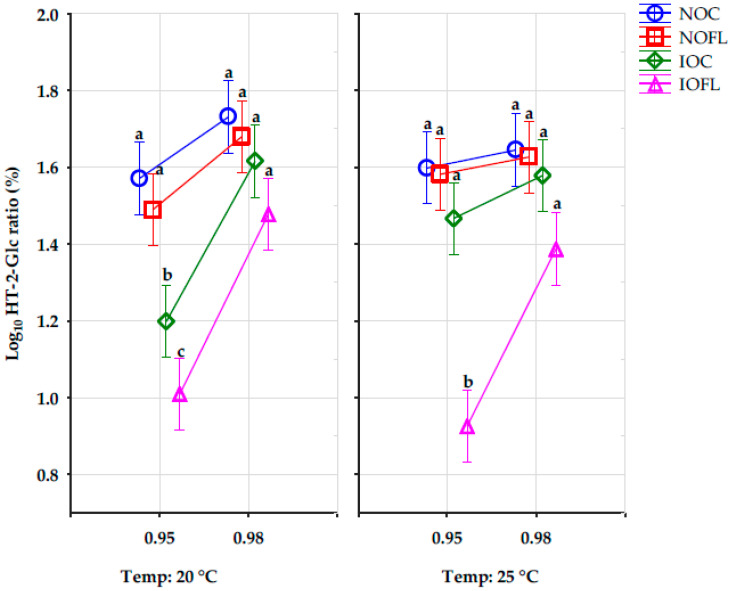
HT-2-Glc concentration ratios (%) in all oat treatments and all storage conditions. Vertical bars denote 0.95 confidence intervals. Different letters show significant differences in HT-2-Glc concentration ratios between all oat treatments at each water activity level for each temperature using the Tukey HSD test. HT-2-Glc: HT-2-glucoside. Water activity: 0.95 and 0.98. Temperature: 20 and 25 °C. NOC: naturally contaminated oat control. NOFL: naturally contaminated oat + *F. langsethiae*. IOC: irradiated oat control. IOFL: irradiated oat + *F. langsethiae*. Concentrations of analytes <LOD and <LOQ are assigned with values of LOD/2 and LOQ/2, respectively [35].

The effect of the a_w_, T, and the combined interactions of the storage conditions and the different treatments on the concentration ratios (%) of HT-2-Glc are significant, except for the interaction between treatment and T, as well as treatment, a_w_, and T, as shown in Figure 1 below. The different letters of the Tukey HSD test show significant differences in the HT-2-Glc concentration ratios between both water activities at each temperature for all treatments. The HT-2-Glc concentration ratios in both irradiated treatments (IOC and IOFL) at 0.95 a_w_ at 20 °C were significantly different from other treatments, while at 25 °C, HT-2-Glc concentration ratios in the irradiated inoculated oats (IOFL) at 0.95 a_w_ were significantly different from other treatments.

The percentage concentration ratios of HT-2-Glc were calculated using Equation (1).

HT-2-Glc % ratio = [(HT-2-Glc)/(T-2 + HT-2 + HT-2-Glc)] × 100(1)

### 2.5. Impact of Water Activity, Temperature, and Treatments on DAS Concentrations

**Figure 2:**
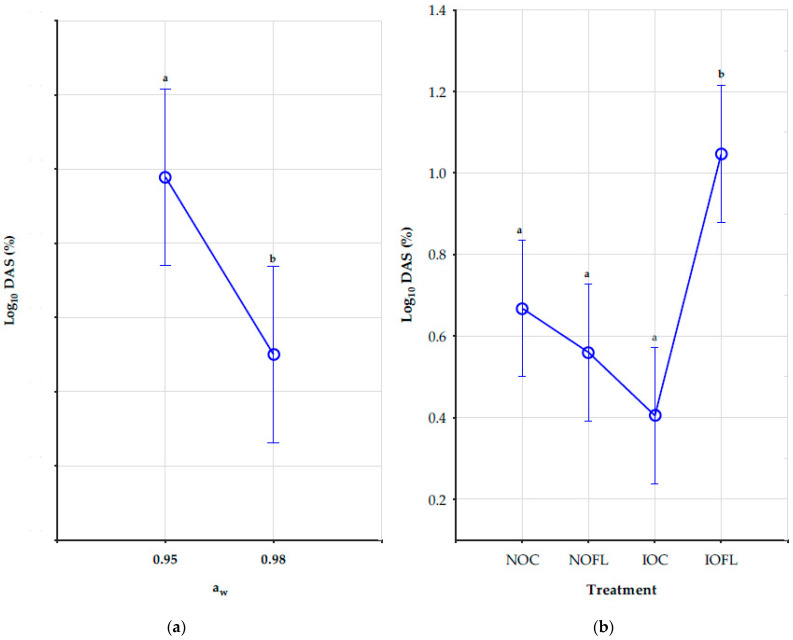
Impact of (**a**) water activity and (**b**) treatment on DAS concentrations (ng/g). Vertical bars denote 0.95 confidence intervals. Different letters show significant differences in DAS concentrations between both water activities and among the treatments using the Tukey HSD test. DAS: diacetoxyscirpenol. a_w_: water activity (0.95 and 0.98). NOC: naturally contaminated oat control. NOFL: naturally contaminated oats + *F. langsethiae*. IOC: irradiated oat control. IOFL: irradiated oat + *F. langsethiae*. Concentrations of analytes <LOD and <LOQ are assigned with values of LOD/2 and LOQ/2, respectively [35].

Interactions between a_w_, T, and treatment have no significant impact on the concentrations (ng/g) of DAS. However, water activity and treatment as a single factor had a significant effect on DAS concentrations, as shown in Figure 2a,b. Different letters of the Tukey HSD test show significant differences in DAS concentrations between both water activities and in each treatment. DAS concentrations decreased significantly as water activities increased from 0.95 a_w_ to 0.98 a_w_ in all treatments. Its concentrations were highest at 0.95 a_w_ at 20 °C for both natural treatments. Although there was a decrease in DAS concentrations with temperature rise, the effect of temperature was not significant in all treatments. Also, DAS concentrations in all treatments and all conditions were not significantly different, except for concentrations in the irradiated inoculated oats (IOFL), as shown in Figure 2b. The mean concentration values of DAS in all treatments and conditions are shown in Table S4 in the Supplementary Materials.

### 2.6. Impact of a_w_ and Temperature on DON and DON-3-G in Both Natural and Irradiated Oat Grains with and without Fusarium langsethiae Inoculation

In the naturally contaminated oat control samples at 0.98 a_w_ and both temperatures, the levels of DON-3-G were about 2.7 times lower than the DON levels. It was present at levels high enough to increase the total DON content in the oats, exceeding the legislative limits. Surprisingly, DON-3-G concentrations at 0.98 a_w_ and 20 °C had a high mean value of 1869 µg/kg, which, in context, is higher than the maximum limits for DON. DON and DON-3-G concentrations increased with the rise in a_w_ but decreased with the temperature rise. However, significant differences in concentrations were only observed at 0.98 a_w_ and 25 °C, as shown in Table 3 below.

Meanwhile, in the naturally contaminated oats + *F. langsethiae*, DON-3-G and DON concentrations significantly increased with an increase in water activity. Also, there was a significant increase in DON-3-G concentrations with an increase in temperature at 0.98 a_w_. In both irradiated oat treatments, there were no significant differences in the concentrations of DON-3-G and DON as the water activity and temperature increased (Table 3).

The *p* values of the statistical differences in the concentrations of DON-3-G/DON under different storage conditions in all oat treatments are shown in Table S5 in the Supplementary Materials.

### 2.7. Impact of a_w_ and Temperature on the Concentrations of Other Secondary Metabolites and Emerging Mycotoxins in Natural and Irradiated Oat Grains with and without Fusarium langsethiae Inoculation

In the naturally contaminated oat control samples, the highest concentrations of nivalenol were observed at 0.95 a_w_ at 25 °C, with a mean value of 230 ng/g. Also, 3-acetyldeoxynivalenol (3-AcDON) and 15-acetyldeoxynivalenol (15-AcDON) concentrations increased as water activity increased but decreased with the temperature rise. The enniatin (A, A1, B, B1), beauvericin (BEA), and moniliformin (MON) concentrations mostly increased with a rise in water activity and temperature, with all mycotoxins having the highest concentrations at 0.98 a_w_ at 25 °C.

In the naturally contaminated oats + *F. langsethiae*, the enniatins were below the limit of quantitation (LOQ) at 0.95 a_w_, but their concentrations almost doubled at 0.98 a_w_ with the temperature rise. Generally, the concentrations of most emerging mycotoxins fell below the LOD and LOQ in the irradiated oat control and irradiated inoculated oats, respectively (Table 4).

## 3. Discussion

There is limited information available on the levels of T-2, HT-2, and HT-2-Glc in oats that are subjected to various storage conditions. This study aims to provide a detailed report on the concentrations of T-2, HT-2, HT-2-Glc, DON, DON-3-glucoside, and DAS (for the first time) in naturally and *Fusarium langsethiae* contaminated oats. naturally *Fusarium langsethiae*-contaminated under different environmental conditions. The chosen storage conditions were 0.95 and 0.98 a_w_ at temperatures of 20 and 25 °C, which are conducive to the growth of *Fusarium* species and mycotoxin contamination in oat grains. These temperatures are similar to those found in the European climate [36]. Previous research has indicated that *Fusarium langsethiae* may colonize oat grains before and after flowering due to specific environmental factors, leading to the accumulation of HT-2 and T-2 [37]. Values of 0.95–0.98 a_w_ may represent a moisture content between 25 and 28% [38] that can be achieved in oat grains post-harvest due to inefficient drying or compromised storage conditions resulting from pest infestations, silo leaks, seasonal rainfall, or temperature changes [39].

The influence of a_w_ and temperature on the levels of HT-2-Glc and HT-2 toxin in naturally contaminated oats (control samples) was found to be insignificant. However, the highest concentrations of these toxins were observed at 0.95 a_w_ and 20 °C, indicating that *Fusarium langsethiae* was able to grow well in grains under these conditions. This finding contrasts with a previous study which reported that the highest growth range for *F. langsethiae* in vitro (oat-based medium) occurred at 0.98–0.995 a_w_ and 25 °C [31]. It is understood that *F. langsethiae* will continue to thrive under cool and damp conditions. Interestingly, under the same storage conditions (0.95 a_w_ and 20 °C) and treatment (naturally contaminated oats control), the concentration of HT-2-Glc was approximately 1.5 times higher than that of HT-2. The levels of T-2 were below the LOQ compared to its acetylated form, HT-2 toxin. Other studies have also reported higher concentrations of HT-2 than its precursor, T-2 toxin [2,4]. Daud et al. [40] reported that concentrations of HT-2 toxins were higher than T-2 toxins in all conventional and organic Scottish oats examined. However, the interactions between different abiotic factors were not considered in these previous studies. Medina and Magan [41] reported higher concentrations of T-2 toxin than HT-2 toxin at 0.95 a_w_ and 20 °C produced by *F. langsethiae* strains isolated from English oats grown on an oat-based medium. The contrast to the T-2 concentrations reported in our study could be due to the deacetylation of T-2 toxin to HT-2 toxin by the oat grains, which did not occur in the oat-based media. Since studies on the metabolic pathways of HT-2 in *F. langsethiae* are limited, there are still uncertainties about whether the high production of HT-2 was mainly due to the strain of fungus present or the natural mycobiota rapidly hydrolysing T-2 to HT-2 due to ecological stress, or the cereal itself as reported in other studies by Lattanzio et al. [42] and Kokkonen et al. [43]. Weather conditions in different geographical locations could also be responsible for differences in toxin contamination [37,44].

As the population of *Fusarium langsethiae* increased due to inoculation, the concentrations of T-2 and HT-2 were found to be below the LOD or LOQ under all storage conditions. This indicates that the growth and toxin production of *F. langsethiae* may be affected by other competitive fungi that are present in the oat grains.

The irradiated inoculated oats had a higher concentration of *F. langsethiae* spores (10^6^ spores/mL) compared to the naturally contaminated oats + *F. langsethiae* samples. This was due to the absence of or lesser competition from other natural fungi that compete for space and nutrients. The concentrations of T-2, HT-2, and HT-2-Glc were high in the irradiated inoculated oat grains and exceeded the recommended levels set by the European Union under all storage conditions. This demonstrates the *F. langsethiae* strain’s ability to produce T-2, HT-2, and its glucoside HT-2-Glc under both water and temperature stress. The concentrations of T-2, HT-2, and HT-2-Glc were significantly different in most storage conditions, except at 0.98 a_w_ and 20 °C.

Generally, the highest concentrations of HT-2-Glc were observed at 0.98 a_w_ and 20 °C in both irradiated oat treatments. A similar observation was made by Mylona and Magan [32], who reported the highest concentrations of T-2 and HT-2 toxins in irradiated oat samples at a temperature of 25 °C and a water activity of 0.97 a_w_ after ten days of storage. The study revealed that water activity had a greater impact on toxin production than temperature. This finding supports our observations in the irradiated oat grains.

Although there are currently no legal or recommended limits for diacetoxyscirpenol (DAS) in oats, the naturally contaminated oat control samples showed the highest concentrations of DAS at 0.95 a_w_ and a temperature of 20 °C, with a mean value of 12 µg/kg. However, DAS levels decreased in the naturally contaminated oats + *F. langsethiae*, despite the fungal inoculation. The highest concentrations of DAS were observed at 0.95 a_w_ and a temperature of 20 °C, with a mean value of 7 µg/kg. On the other hand, the irradiated oats under the same storage conditions had a mean value of 19 µg/kg, as shown in Table S4 of the Supplementary Materials. These findings suggest 20 °C as the optimum temperature for DAS production. Bryła et al. [45] analysed DAS alongside other trichothecenes in oats and reported its concentrations to be lower than the limit of quantitation (LOQ) of 1 μg/kg, which was lower than the LOQ in our study. Also, it is important to note that the study did not consider DAS under different environmental conditions.

As a regulated mycotoxin, it is important to analyse DON concentrations alongside T-2 and HT-2 toxins in oats. Our findings show that the concentrations of DON were higher than T-2 and HT-2 toxin concentrations in the naturally contaminated oats. This agrees with the observations of Nathanail et al. [46]. Their study reported DON and its glucoside, DON-3-G, with mean values of 2690 µg/kg and 806 µg/kg, respectively, while T-2, HT-2, and its glucoside HT-2-Glc had mean concentrations of 60.1 µg/kg, 159 µg/kg, and 41.4 µg/kg, respectively. However, the Finnish oat samples used had a moisture content below 15% (<0.70 a_w_) compared to the wetter conditions used in our study. These studies suggest that *Fusarium graminearum* might have a growth advantage over *Fusarium langsethiae* under the conditions studied, leading to an increase in the production of DON in the oats, with the occurrence of DON in oats reported by other studies [6,47].

In contrast to our findings, Langseth and Rundberget [2] reported higher concentrations of HT-2 (115 µg/kg) and T-2 (60 µg/kg) than DON concentrations, which were 56 µg/kg in Norwegian oats samples. The mean value of the moisture content of the Norwegian oats was 15 (±2). In contrast to the naturally contaminated oat control samples, DON concentrations in the irradiated oats were relatively low due to the increased concentrations of T-2 and HT-2 toxins produced by *F. langsethiae*.

Surprisingly, DON and DON-3-G concentrations were found in the irradiated control samples in the wettest condition, 0.98 a_w_, at both temperatures. This suggests that the moisture content plays a crucial role in the fungal contamination of grains.

In the naturally contaminated oat control samples, enniatin B had the highest mean concentrations (539 µg/kg) among all enniatins in all storage conditions at 0.98 a_w_ at 25 °C. The occurrence of enniatin B at the highest concentrations among all the enniatins studied was also reported by Uhlig et al. [48]. The mean concentrations of enniatin B in their study were 47 µg/kg, 490 µg/kg, and 790 µg/kg in Norwegian oats, barley, and wheat samples, respectively. However, the grains analysed did not have the same storage conditions as those used in our study. In the wettest and warmest storage conditions, MON and BEA had their highest mean concentrations of 3050 µg/kg and 834 µg/kg, respectively. However, in the naturally contaminated oats + *F. langsethiae*, MON and BEA concentrations were remarkably lower at the same storage conditions (Table 4).

## 4. Conclusions

This research shows that the levels of *Fusarium* toxins vary based on different storage conditions. As the water activity changes, conjugated mycotoxins co-occur with their precursor toxins, other secondary metabolites, and emerging toxins in stored oats. Although the amount of T-2, HT-2, and HT-2-Glc in the naturally contaminated oats was not higher than the recommended or indicative levels, the coexistence of DON-3-G with DON was significant, leading to an increase in the total deoxynivalenol content and thus increasing the overall toxicity of the oats.

Mycotoxin levels in the inoculated grains indicated a high risk of contamination. This risk could occur due to inefficient drying regimes or changes in the storage environment, especially if the populations of *Fusarium langsethiae* are high in oats. These findings highlight the importance of conducting a robust analysis of mycotoxins in grains to avoid underestimating the total toxin content. Furthermore, since the toxicity of T-2 and HT-2 is similar [49,50] and our findings showed no significant differences in their concentrations, it is important to efficiently dry grains after harvest and monitor the abiotic conditions of the storage environment. This will help to reduce high concentrations of either individual or sums of trichothecene toxins and their respective conjugates in oat grains.

## 5. Materials and Methods

### 5.1. Fungal Isolates

*Fusarium langsethiae* (2390/2391), known to produce T-2 and HT-2 toxins, was used in this study. The strain was maintained in glycerol/water (70:30, *v*/*v*) at −20 °C in the culture collection of the Applied Mycology Group, Cranfield University. *Fusarium langsethiae* was grown on malt extract agar (MEA) + chloramphenicol (anti-bacterial agent) incubated at 25 °C for 7 days and then sub-cultured on V8 agar at 25 °C for 7 days for active sporulation.

### 5.2. Oat Grains and Moisture Adsorption Curve Analysis

Oat grains (with husk) were collected from Bedfordshire farms (2019 harvest). Grains were stored at 4 °C ten months before the experiment, where 5 kg of grains was exposed to 12.5–15 kGys (STERIS, Bradford, UK) to reduce microbial contamination from the grains while retaining their germination capacity. The initial mycobiota of the natural and irradiated oats were analysed by placing five grains equidistant on five MEA+ media on 9 cm Petri plates in a sterile flow bench incubated at 25 °C for 7 days. The fungal identification was visually evaluated with a stereoscope.

In total, 10.0 g of both treated oat grains both oats and treated grains was placed in 25 mL universal glass bottles and a known amount of water (0.1–3.5 mL) was added to the grains. The bottles were tightly sealed to reduce moisture loss and stored at 4 °C for 24 h with regular shaking. The samples were equilibrated at room temperature for 1 h and analysed for water activity with a water activity meter (4 TE Decagon devices, Aqualab Inc., Pullman, WA, USA) and moisture content, with the samples oven-dried at 105 °C overnight. Three replicates were analysed for each grain treatment. The amount of added water was plotted against a_w_ values to modify the grains to the targeted a_w_ levels. The relationship between the moisture content (MC) (dry weight basis) and a_w_ values was also plotted and noted as discussed by Chulze et al. [38].

### 5.3. Grain Inoculation

Spores were harvested aseptically from a 7-day-old *F. langsethiae* plate scraped with a sterile spatula with sterile Tween 80 water (0.05% *v*/*v*). Then, 10 mL of the suspension was transferred into a sterile tube and shaken to obtain a homogenous mix. The spores’ concentrations were counted and calculated with a Thoma cell counting chamber. The spore suspension was further diluted with Tween 80 water to achieve the targeted concentrations of 10^6^ spores/mL. Targeted a_w_ levels of 0.95 and 0.98 a_w_ for the natural and irradiated oat grains were calculated using the moisture adsorption curve. A total of 120 g of grains was mixed with sterile water (1 mL less for spore inoculum) and stored at 4 °C for 24 h to equilibrate. The control treatments were also modified with sterile water. The equilibrated grains were dispensed (15 g) into 40 mL clear glass volatile organic analysis (VOA) vials with sealable polytetrafluoroethylene (PTFE) caps containing a silicone septum for gas exchange. Grains with the same a_w_ levels were placed in 12 L poly-propylene environmental chambers with 2 × 500 mL beakers of glycerol–water solution to maintain the target equilibrium relative humidity (ERH) of the atmosphere for each a_w_ level. The chambers were stored at temperatures of 20 and 25 °C for 17 days. The grains were dried at 55 °C overnight, ground, and stored at −20 °C before further analysis.

### 5.4. Mycotoxin Analysis

#### 5.4.1. Chemical Reagents

HT-2-toxin-3-O-β-D-glucoside was synthesized by Michlmayr et al. [51]. Other mycotoxin standards supplied by the Institute for Global Food Security, Queen’s University Belfast (QUB), UK were purchased from Romer Labs (Tulln, Austria) They include DON-3-G, HT-2 toxin, T-2 toxin, DON, 15-acetyl-deoxynivalenol (15-AcDON), 3-acetyl-deoxynivalenol (3-AcDON), DAS, enniatin A (ENN A), enniatin A1 (ENN A1), enniatin B (ENN B), enniatin B1 (ENN B1), moniliformin (MON) beauvericin (BEA), and nivalenol (NIV). LC-MS/MS-grade methanol, acetonitrile, and formic acid (Honeywell, Seelze, Germany); ammonium acetate (MS grade, Sigma-Aldrich, Darmstadt, Germany); and glacial acetic acid (Sigma-Aldrich, Burlington, MA, USA) were used. Water was purified successively by reverse osmosis and using a Milli-Q plus system from Millipore (Merck, Molsheim, France).

#### 5.4.2. Sample Preparation and Extraction

The initial mycotoxin concentrations of the oat grains were analysed. Following the storage experiment, 64 contaminated oat samples (including replicates) were analysed for mycotoxins. A volume of 4 mL of extraction solvent (ACN/H_2_O/acetic acid, 79:20:1, *v*/*v*/*v*) was added to 1.00 g of ground oat grains. Extraction was carried out for 90 min using a multitube vortex (VWR DVX-2500, VMR International Ltd. Leicestershire, UK), followed by centrifugation for 15 min at 5000 rpm on a Rotina 380R centrifuge (Hettich, Tuttlingen, Germany). Then, 200 µL of the extract was diluted with 800 µL of dilution solvent (ACN/H_2_O/acetic acid, 20:79:1, *v*/*v*/*v*). The diluted extracts were then filtered into LC-MS/MS vials using a 0.22 µm PTFE filter, with 5 µL of the diluted extract injected into the LC-MS/MS system for analysis.

#### 5.4.3. LC-MS/MS Parameters

The LC-MS/MS machine used in this study was a LQTRAP 5500+ MS/MS system (SCIEX, Framingham, MA, USA) equipped with a Turbo V electrospray ionization (ESI) source, coupled to an ExionLC AD System (SCIEX, Framingham, MA, USA). Chromatographic separation was performed at 27 °C on a Gemini C_18_-column, 100 × 4.6 mm (Phenomenex, Torrance, CA, USA). The chromatographic method, as well as chromatographic and mass spectrometric parameters, were adapted from Malachová et al. [52]. Liquid chromatography–tandem mass spectrometry (LC-MS/MS) (SCIEX, Framingham, MA, USA) was performed in the time-scheduled MRM mode both in positive and negative polarities in one chromatographic run per sample by scanning two fragmentation reactions per analyte. Elution was carried out in binary gradient mode. Both mobile phases contained 5 mM of ammonium acetate and were composed of water/methanol/acetic acid at 89:10:1 (*v*/*v*/*v*; eluent A) and 2:97:1 (*v*/*v*/*v*; eluent B), respectively, with a sample injection volume set at 5 µL, with a total runtime of 7.5 min. The gradient elution program for the elution of mycotoxins was as follows: 0 min 5% B, 0.5 min 5% B, 2.5 min 70% B, 3.5 min 95% B, 5 min 95% B, 7.5 min 5% B. The mass spectrometry parameters used are outlined in Table 5.

#### 5.4.4. Optimised LC-MS/MS Method Validation

The optimised LC-MS/MS method for the analysis of mycotoxins in oats was validated based on the acceptable performance criteria of analytical methods set and updated by European Commission regulation No. 2021/808/EC [32]. The performance characteristics evaluated were linearity (r^2^), limit of detection (LOD), limit of quantification (LOQ), matrix effect or the signal suppression/enhancement (SSE), recovery of the extraction process (RE), absolute recovery (R_A_), and repeatability. Extraction and apparent recoveries were determined from five different lots of oats spiked in quintuplicate on three separate days, with the multi-mycotoxin working standard solution.

A working standard of 0.5 µg/mL (for DON-3-G and the enniatins) and at 1 µg/mL (for other toxins listed in Section 5.4.1) was prepared from the individual intermediate standards, diluted with ACN/H_2_O (50:50, *v*/*v*) to achieve the desired concentration of each analyte in the working standard. Quantification was performed via external calibration using an eight-point calibration curve achieved by serial dilutions of the multi-analyte working standard solution. Data were further processed using Analyst^®^ 1.7.1 and SCIEX OS-Q 3.0. The validation procedure was adapted from Siri-Anusornsak et al. [53].

The apparent recovery (R_A_), matrix effect (SSE), and extraction efficiency (RE) were calculated using Equations (2)–(4) below.

R_A_ (%) = area (sample spiked before extraction)/area (neat solvent standard) × 100 (2)

SSE (%) = area (sample spiked after extraction)/area (neat solvent standard) × 100(3)

RE (%) = area (sample spiked before extraction)/area (sample spiked after extraction) × 100(4)

### 5.5. Statistical Analyses

JMP^®^ Pro 16 and Statistica 14.0.1 software were used for data analysis. Data were tested for normality and homoscedasticity using the Shapiro–Wilk and Levene tests, respectively. When data failed the normality test, data were transformed to achieve normality. Transformed data were normally distributed; therefore, a one-way ANOVA was used to find differences between groups. Also, nonparametric comparisons for each pair using the Wilcoxon method showed differences among each toxin pair for each treatment. A Tukey HSD test was used to analyse the significant impact of the interactions of the storage conditions and treatments on the DAS and HT-2-glucoside concentration ratios. The linearity assumption and the normal distribution of residuals were examined, resulting in normal plots of the residuals. Concentrations of analytes <LOD and <LOQ are assigned with values of LOD/2 and LOQ/2, respectively, for the calculation of the mean concentration and statistical analysis [35]. Statistical analyses performed were considered significant when *p* values were <0.05.

## Figures and Tables

**Table 1:** Mean values (*n* = 75) of the inter-day precision within-lab reproducibility (WLR) method validation for wheat grains.

Components	RSD (%)	R_A_ (%)	SSE (%)	RE (%)	LOD (ng/g)	LOQ (ng/g)
Moniliformin	6	87	116	74	2.0	6.7
15-Acetyldeoxynivalenol	13	57	65	95	2.8	9.2
3-Acetyldeoxynivalenol	3	95	94	99	2.2	7.3
Beauvericin	5	94	92	101	2.6	8.6
Diacetoxyscirpenol	5	93	89	100	1.9	6.4
Deoxynivalenol	3	96	96	101	3.7	12.4
DON-3-Glucoside	5	69	88	77	1.7	5.5
Enniatin A	7	86	84	103	1.0	3.4
Enniatin A1	5	89	91	98	1.6	5.4
Enniatin B	9	97	101	98	5.1	17.0
Enniatin B1	7	94	96	98	2.8	9.2
HT-2 toxin	3	94	96	98	4.1	13.6
HT-2-Glc	5	93	93	95	5.6	15.8
Nivalenol	22	80	85	92	19.0	63.2
T-2-toxin	2	94	94	99	3.4	11.4

R_A_—apparent recovery, RE—extraction efficiency, RSD—relative standard deviation, SSE—signal suppression/enhancement, LOD—limit of detection, LOQ—limit of quantitation. *n* = average values of 75 replicates from the 5 different lots of oats.

**Table 2:** Influence of a_w_ and T on the concentrations of T-2, HT-2, and HT-2-Glc in oats.

Mycotoxins (ng/g of Grains) ^a^									
		20 °C				25 °C			
Treatments	a_w_	T-2 ^1^	HT-2 ^2^	HT-2-Glc ^3^	Sum	T-2 ^1^	HT-2 ^2^	HT-2-Glc ^3^	Sum
Naturally contaminated oat control	0.95	<LOQ	26	41	67	<LOD	<LOD	<LOD	-
	0.98	<LOD	<LOD	<LOQ	-	<LOD	<LOD	<LOQ	-
Naturally contaminated oat + *F. langsethiae*	0.95	<LOQ	<LOQ	<LOQ	-	<LOD	<LOQ	<LOD	-
	0.98	<LOQ	<LOQ	37	37	<LOD	<LOQ	<LOQ	-
Irradiated oat control	0.95	<LOQ	20	<LOD	20	<LOQ	<LOQ	<LOQ	-
	0.98	<LOQ	<LOQ	<LOD	-	<LOQ	<LOQ	<LOD	-
Irradiated oat + *F. langsethiae*	0.95	4488 *	1655 *	704 *	6847	1822 *	645 *	226 *	2693
	0.98	7152	6037	5925	19,114	4117	7167	3690	14,974

^a^ Results are a mean of 4 replicates in different storage conditions. a_w_—water activity. T—temperature. ^1^ T-2 toxin, ^2^ HT-2 toxin, ^3^ HT-2-glucoside. * = significant differences in the concentrations of T-2 and HT-2 at the same a_w_ (for the same temperature in each row). <LOD—below limit of detection. <LOQ—below limit of quantitation. Sum—T-2 + HT-2 + HT-2-Glc.

**Table 3:** Influence of a_w_ and T on the concentrations of deoxynivalenol and DON-3-glucoside in oats.

Mycotoxins (ng/g of Grains) ^a^							
		20 °C			25 °C		
Treatments	a_w_	DON ^1^	DON-3-Glc ^2^	Sum	DON ^1^	DON-3-Glc ^2^	Sum
Naturally contaminated oat control	0.95	72	18	90	27	6	33
	0.98	5137	1869	7006	2212 *	818 *	3030
Naturally contaminated oat + *F. langsethiae*	0.95	1141 *	58 *	1199	1636 *	64 *	1700
	0.98	3995 *	1061 *	5056	5052 *	2297 *	7349
Irradiated oat control	0.95	<LOD	<LOD	<LOD	<LOD	<LOD	<LOD
	0.98	21 *	7 *	28	33 *	8 *	41
Irradiated oat + *F. langsethiae*	0.95	20	<LOQ	20	14	<LOQ	14
	0.98	55 *	7 *	62	16 *	7 *	23

^a^ Results are a mean of 4 replicates in different storage conditions. a_w_—water activity. T—temperature. ^1^ Deoxynivalenol, ^2^ Deoxynivalenol-3-Glucoside. * = significant differences in the concentrations of DON and DON-3-G at the same a_w_ (for the same temperature in each row). <LOD—below limit of detection. <LOQ—below limit of quantitation. Sum—DON + DON-3-G.

**Table 4:** Influence of a_w_ and T on the concentrations of other secondary metabolites and emerging mycotoxins in oats.

Mycotoxins (ng/g of Grains) ^a^											
Treatments	T (°C)	a_W_	3-AcDON	15-AcDON	NIV	ENN A	ENN A1	ENN B	ENN B1	MON	BEA
Naturally contaminated oat control	20	0.95	27	29	137	<LOQ	<LOQ	19	13	9	29
		0.98	89	126	138	8	56	251	192	205	142
	25	0.95	10	29	230	<LOQ	6	19	17	63	51
		0.98	26	89	90	25	190	539	461	3050	834
Naturally contaminated oat + *F. langsethiae*	20	0.95	9	62	237	<LOQ	<LOQ	<LOQ	<LOQ	13	23
		0.98	26	192	860	<LOQ	9	45	29	<LOQ	141
	25	0.95	21	93	206	<LOQ	<LOQ	<LOQ	<LOQ	19	22
		0.98	92	172	610	<LOQ	20	88	62	21	142
Irradiated oat control	20	0.95	<LOD	12	38	<LOD	<LOD	<LOD	13	<LOD	4
		0.98	<LOD	15	42	<LOD	<LOD	<LOD	13	<LOD	12
	25	0.95	<LOD	27	55	<LOD	<LOD	<LOD	24	<LOD	15
		0.98	<LOD	33	60	<LOD	<LOD	<LOD	21	<LOD	22
Irradiated oat + *F. langsethiae*	20	0.95	<LOQ	44	70	<LOQ	<LOQ	<LOQ	<LOQ	<LOQ	25
		0.98	<LOQ	68	90	<LOQ	<LOQ	<LOQ	<LOQ	<LOD	47
	25	0.95	<LOQ	47	147	<LOQ	<LOQ	<LOQ	12	<LOD	36
		0.98	<LOQ	81	<LOQ	<LOQ	<LOQ	<LOQ	12	<LOD	39

^a^ Results are a mean of 4 replicates in different storage conditions. a_w_—water activity. T—temperature. AcDON—Acetyldeoxynivalenol. NIV—Nivalenol. ENN—Enniatin. MON—Moniliformin. BEA—Beauvericin. <LOD—below limit of detection. <LOQ—below limit of quantitation.

**Table 5:** Optimised MS/MS parameters for the analysed mycotoxins, including precursor ions, product ions, declustering potential (DP), collision energy (CE), and collision cell exit potential (CXP).

Mycotoxins	Precursor Ion (*m*/*z*)	Product Ion (*m*/*z*)	DP (V)	CE (V)	CXP (V)
Regulated mycotoxins					
Deoxynivalenol	297.1	249.1	91	21	20
	297.1	203.2	91	21	20
T-2 toxin	484.3	215.2	76	29	18
	484.3	185.1	76	31	11
HT-2 toxin	442.3	263.1	71	19	14
	442.3	215.1	71	19	22
Masked Mycotoxins					
3-Acetyldeoxynivalenol	397.3	59.2	−60	−38	−8
	397.3	307.1	−60	−20	−7
15-Acetyldeoxynivalenol	339.1	321.3	81	13	18
	339.1	261.1	81	17	14
Deoxynivalenol-3-Glucoside	517.2	427.1	−115	−30	−11
	517.2	457.2	−115	−20	−19
HT-2-Glc	604.3	323.1	101	17	16
	604.3	263.1	101	23	14
Emerging mycotoxins					
Beauvericin	801.3	784.3	141	27	14
	801.3	244.1	141	43	12
Diacetoxyscirpenol	384.2	307.3	86	17	16
	384.2	247.3	86	21	14
Enniatin A	699.4	682.4	10	27	24
	699.4	210.2	10	39	22
Enniatin B	657.3	640.3	10	27	22
	657.3	196.1	10	39	10
Enniatin B_1_	671.3	654.4	6	27	22
	671.3	196.1	6	41	22
Enniatin A_1_	685.4	668.5	11	27	12
	685.4	210.1	11	39	10
Moniliformin	96.9	41.2	−5	−38	−14
Nivalenol	371.1	281.1	−90	−20	−15
	371.1	59.1	−90	−50	−7

## Data Availability

Data supporting this study are included within the article and Supplementary Materials.

## Supplementary Materials

The following supporting information can be downloaded at https://www.mdpi.com/article/10.3390/toxins16040166/s1: Table S1: The measurement uncertainty for each analyte. Table S2: Statistical differences in the concentrations of each trichothecene at all storage conditions using one-way ANOVA and nonparametric comparisons for each pair using the Wilcoxon method. Table S3: Statistical differences in the concentrations of trichothecenes at each water activity level and temperature for all treatments using one-way ANOVA and nonparametric comparisons for each pair using the Wilcoxon method. Table S4. Influence of a_w_ × T on the concentrations of diacetoxyscirpenol in oats. Table S5: Statistical differences in the concentrations of DON-3-G to DON at each water activity level and temperature for all treatments using one-way ANOVA and nonparametric comparisons for each pair using Wilcoxon method.
