# Supplementary material for: Impact of Environmental Conditions on the Concentrations of Trichothecenes, Their Glucosides, and Emerging Fusarium Toxins in Naturally Contaminated, Irradiated, and Fusarium langsethiae Inoculated Oats
Source: Toxins (Basel). 2024 Mar 22;16(4):166. doi: 10.3390/toxins16040166 (PMC11055103; doi:10.3390/toxins16040166)
Supplement: Supplementary file 1 [file toxins-16-00166-s001.zip › toxins-2883991-supplementary.pdf]

Supplementary materials: Impact of Environmental Conditions on the Concentrations of Trichothecenes, Their Glucosides, and Emerging Fusarium Toxins in Naturally Contaminated, Irradiated, and Fusarium langsethiae Inoculated Oats

Abimbola Oluwakayode, Brett Greer, Julie Meneely, Franz Berthiller, Rudolf Krska, and Angel Medina

Table S1: The measurement uncertainty for each analyte

**Analytes**	**2SD**	**3SD**	**Mean Conc. (µg/kg)**	**U_r (%)**	**U (%)**
Moniliformin	22.2	33.3	174	13	19
15-Acetyldeoxynivalenol	30.2	45.2	114	26	40
3-Acetyldeoxynivalenol	10.1	15.1	190	5	8
Beauvericin	10.2	15.3	93.8	11	16
Diacetoxyscirpenol	20.0	30.0	187	11	16
Deoxynivalenol	12.3	18.5	191	6	10
DON-3-G	6.5	9.7	69.0	9	14
Enniatin A	11.5	17.3	85.9	13	20
Enniatin A_1	9.5	14.3	88.8	11	16
Enniatin B	16.8	25.2	97.5	17	26
Enniatin B_1	13.4	20.1	93.8	14	21
HT-2 toxin	9.6	14.5	188	5	8
HT-2-Glc	9.9	14.9	192	5	8
Nivalenol	71.7	108	160	45	67
T-2 toxin	9.0	13.4	187	5	7

SD- Standard Deviation. Conc.- Concentration.

Ur: Relative expanded measurement uncertainty estimated from intra-laboratory validation (intermediate precision) data based on five lots of oats, close to a 95% confidence interval.

U: Relative expanded measurement uncertainty estimated from intra-laboratory validation (intermediate precision) data based on five lots of oats, close to a 99% confidence interval.

Table S2: Statistical differences in the concentrations of each trichothecene at all storage conditions using one-way ANOVA and nonparametric comparisons for each pair using the Wilcoxon method.

Treatments		Levels	T-2 toxin	HT-2 toxin	HT-2-Glc	DON	DON-3-G	DAS
Naturally contaminated oat control	a_w	One-way ANOVA	0.3660	0.2225	0.6449	0.0009	0.0015	0.0198
		0.98- 0.95	0.6721	0.3184	0.9575	0.0009	0.0009	0.0135
	T [°C]	One-way ANOVA	0.4929	0.2784	0.3600	0.2483	0.2758	0.2031
		25- 20	0.2237	1.0000	0.7899	0.3717	0.3184	0.4005
Naturally contaminated oat + *F. langsethiae*	a_w	One-way ANOVA	0.2930	0.3516	0.3834	<.0001	0.0001	0.5608
		0.98- 0.95	0.1036	0.5635	0.3706	0.0009	0.0009	0.3184
	T [°C]	One-way ANOVA	0.547	0.2673	0.2120	0.4350	0.2386	0.4931
		25- 20	0.0661	0.7132	0.6355	0.4948	0.4309	0.6365
Irradiated oat control	a_w	One-way ANOVA	0.0015	<.0001	0.0072	0.0003	<.0001	0.1489
		0.98- 0.95	0.0122	0.0008	0.0116	0.0004	0.0004	0.1709
	T [°C]	One-way ANOVA	0.1362	0.5863	0.0383	0.5082	0.9093	0.9914
		25- 20	0.2202	0.7091	0.1071	0.7791	0.9553	1.0000
Irradiated oat + *F. langsethiae*	a_w	One-way ANOVA	0.0662	<.0001	<.0001	0.2383	0.0422	0.9257
		0.98- 0.95	0.0831	0.0009	0.0009	0.2271	0.0831	0.4309
	T [°C]	One-way ANOVA	0.0307	0.9702	0.3185	0.1389	0.6538	0.0337
		25- 20	0.0313	0.6365	0.2271	0.0074	0.9581	0.1036

DON: Deoxynivalenol. DON-3-G: Deoxynivalenol-3-Glucoside. DAS: Diacetoxyscirpenol. HT-2-Glc: HT-2-glucoside. a_w: water activity. T: Temperature.

Table S3: Statistical differences in the concentrations of trichothecenes at each water activity and temperature for all treatments using one-way ANOVA and nonparametric comparisons for each pair using the Wilcoxon method.

		20 °C				25°C			
**Treatments**	**a_w Levels**	**One-way ANOVA**	**T-2: HT2-Glc**	**HT-2: HT2-Glc**	**T-2: HT-2**	**One-way ANOVA**	**T-2: HT2-Glc**	**HT-2: HT2-Glc**	**T-2: HT-2**
Naturally contaminated oat control	0.95	0.5718	1.000	0.4678	0.3123	0.2139	0.3123	0.8852	0.0304
	0.98	0.1418	0.1241	0.3836	0.1241	0.4106	0.4705	0.6650	0.3123
Naturally contaminated oat + *F. langsethiae*	0.95	0.4139	1.0000	0.4705	0.4705	0.1538	0.0304	0.6650	0.0304
	0.98	0.6135	1.0000	1.0000	0.6650	0.1682	0.0304	0.3123	0.1939
Irradiated oat control	0.95	0.0131	0.0131	0.0131	0.0131	0.0131	0.0131	0.0131	0.0131
	0.98	0.5275	0.1832	0.1832	1.0000	0.4426	0.8852	0.3123	0.4705
Irradiated oat + *F. langsethiae*	0.95	0.0009	0.0304	0.0304	0.0304	<.0001	0.0304	0.0304	0.0304
	0.98	0.7799	0.4705	1.0000	0.4705	0.0071	0.6650	0.0304	0.0304

Table S4. Influence of a_w x T on the concentrations of Diacetoxyscirpenol in oats.

Mycotoxins (ng/g of grains) [a]			
**Treatments**	**a_w**	**20 °C**	**25 °C**
Naturally contaminated oat control	0.95	12	8
	0.98	7	< LOD
Naturally contaminated oat + *F. langsethiae*	0.95	7	< LOQ
	0.98	< LOQ	< LOQ
Irradiated oat control	0.95	< LOQ	< LOQ
	0.98	< LOD	< LOD
Irradiated oat + *F. langsethiae*	0.95	19*	9*
	0.98	19*	9*

[a] Results are a mean of 4 replicates at different storage conditions. a_w -water activity. T- temperature.
*= significant differences (using One-way ANOVA) in the concentrations of DAS for both temperatures in each row. < LOD- below limit of detection. < LOQ- below limit of quantitation.

Table S5: Statistical differences in the concentrations of DON-3-G to DON at each water activity and temperature for all treatments using one-way ANOVA and nonparametric comparisons for each pair using the Wilcoxon method.

**Mycotoxins**	**NOC**			**NOFL**		**IOC**		**IOFL**	
	**a_w, T [°C]**	**ANOVA**	**Wilcoxon**	**ANOVA**	**Wilcoxon**	**ANOVA**	**Wilcoxon**	**ANOVA**	**Wilcoxon**
DON-3-G: DON	0.95, 20	0.3049	0.1939	0.0013	0.0304	1.0000	1.0000	0.0008	0.0304
DON-3-G: DON	0.95, 25	0.3709	1.0000	0.0034	0.0304	1.0000	1.0000	0.0024	0.0304
DON-3-G: DON	0.98, 20	0.0760	0.1939	0.0054	0.0304	0.0079	0.0304	0.1241	0.0304
DON-3-G: DON	0.98, 25	0.0031	0.0304	0.0124	0.0304	0.0536	0.0304	0.0080	0.0304

NOC- Naturally contaminated oat control. NOFL: Naturally contaminated oat + *F. langsethiae.* IOC: Irradiated oat control. IOFL: Irradiated oat +*F. langsethiae.* a_w: water activity. T- Temperature. DON: Deoxynivalenol. DON-3-G: Deoxynivalenol-3-glucoside.
